# Effects of Grass Clipping Mulching on Alfalfa Yield and Quality Through Soil Amelioration in Soda Saline–Alkali Soils

**DOI:** 10.3390/plants15213393

**Published:** 2026-11-02

**Authors:** Weiguang Yang, Xiaojia Shi, Ling Li, Nan Wang, Changyuan Wang, Xiangping Liu

**Affiliations:** College of Animal Science and Technology, Heilongjiang Bayi Agricultural University, Daqing 163319, China; yangweiguang2021@byau.edu.cn (W.Y.); shixiaojia15731969113@byau.edu.cn (X.S.); wang77100179@163.com (N.W.); wangchangyuan@byau.edu.cn (C.W.)

**Keywords:** grass clipping mulching, soda saline–alkali soil, alfalfa, soil amelioration, PLS-SEM, forage quality

## Abstract

Salinization is a major constraint to agricultural sustainability in arid and semi-arid regions worldwide. The ameliorative effects of grass clipping mulching on alfalfa (*Medicago sativa* L.) growth and forage quality in soda saline–alkali land in Northeast China and its regulatory mechanisms were studied. The trial used a randomized block design with four mulch treatments: no-mulch control (CK), low-density (LC, 6000 kg ha^−1^, or 6.0 t ha^−1^), medium-density (MC, 12,000 kg ha^−1^, or 12.0 t ha^−1^), and high-density (HC, 18,000 kg ha^−1^, or 18.0 t ha^−1^). Compared with CK, MC achieved the highest soil water content (18.3% vs. 14.9% in CK), the lowest bulk density (1.26 vs. 1.48 g cm^−3^), and the greatest porosity (52.4% vs. 44.2%); pH, electrical conductivity, total salt, and ESP decreased, while organic matter and available nutrients increased. Overwintering survival rose to 89.1% under HC (+9.3%). Net photosynthetic rate, stomatal conductance, and chlorophyll content increased, and first-cut hay yield increased by 27.3% under HC (3938 vs. 3093 kg ha^−1^). Crude protein reached 20.42%, and relative feed value increased from 158.4 to 167.1. Partial least squares structural equation modeling (PLS-SEM) showed that mulching acted mainly through soil nutrient enrichment, thereby enhancing plant physiology and ultimately forage yield and quality. Grass clipping mulching is a cost-effective strategy for saline–alkali soil remediation and sustainable alfalfa production.

## 1. Introduction

Soil salinization is one of the most pressing environmental challenges constraining agricultural sustainability in arid and semi-arid regions worldwide [1]. The Food and Agriculture Organization (FAO) reports that the current global land area affected by salt has reached 1.381 billion hectares, and this area is still expanding due to climate change, unsustainable irrigation practices, and natural pedogenic processes [2]. In China, saline–alkali land covers about 100 million hectares, mainly in North China—specifically Northeast and Northwest—and coastal regions. Among these areas, the soda saline–alkali soils (solonetz) in Northeast China are particularly troublesome [3,4]. They are characterized by high levels of Na_2_CO_3_ and NaHCO_3_, leading to soil pH above 9.0, increased exchangeable sodium percentage (ESP), and severe degradation of soil physical structure due to clay dispersion and reduced aggregate stability [5,6]. These soil conditions create significant osmotic stress and ion toxicity, hindering root growth, nutrient uptake, and plant growth, which is a major obstacle for grassland agriculture and ecological restoration in the region [5].

Conventional strategies for ameliorating soda saline–alkali soils encompass hydrological engineering, including leaching and drainage, chemical amendments such as gypsum, organic acids, and sulfur, as well as the cultivation of salt-tolerant vegetation [7,8]. However, engineering methods are often prohibitively expensive, require substantial water resources, and may lead to secondary salinization of downstream ecosystems [9]. Although chemical amendments act quickly, their prolonged application can disrupt the ecological balance of soil microbes and fail to fundamentally enhance soil structure [10]. Relying exclusively on the salt tolerance of plants for biological remediation frequently results in establishment failures or reduced yields due to the extreme conditions of the soil environment [11,12]. Therefore, there is an urgent need to develop ecologically sound and cost-effective technologies that can simultaneously improve soil conditions and promote plant growth.

Organic mulching, an ancient agronomic practice involving the surface application of plant residues, has attracted renewed attention for saline–alkali soil remediation [13]. By governing the soil hydrothermal regime, mulching materials effectively suppress moisture evaporation, moderate diurnal temperature fluctuations, and thereby constrain the upward migration and surface accumulation of salts [14]; meanwhile, during decomposition, they gradually release organic acids, polysaccharides, and humic substances that neutralize alkalinity, chelate Na^+^, promote the formation of water-stable aggregates, and enhance pore structure [15,16]. In parallel, the carbon and nutrients supplied by decomposing mulch stimulate microbial activity, enhance nutrient cycling, and foster a more resilient soil microenvironment [16]. These physicochemical effects are particularly valuable in soda saline–alkali soils, where high pH and ESP jointly restrict water infiltration, nutrient availability, and root growth [17]. While previous studies have established the salt-reducing and alkali-suppressing benefits of organic mulching in coastal saline soils and secondary salinized croplands [18], the systematic mechanisms by which grass clipping mulching ameliorates soda saline–alkali soils remain inadequately understood.

Alfalfa (*Medicago sativa* L.) is widely regarded as an ideal leguminous forage for saline–alkali land improvement due to its high yield, superior nutritional quality, nitrogen-fixing capacity, and moderate tolerance to saline–alkali stress [19,20]. Nevertheless, under the dual pressure of high pH and elevated ESP characteristic of soda saline–alkali soils, alfalfa seed germination is inhibited, root system development is restricted, and biomass accumulation is limited, preventing the full expression of its production potential [21]. Therefore, improving the rhizosphere soil environment to alleviate saline–alkali stress and promote alfalfa root establishment has become an urgent technical challenge for alfalfa cultivation in these regions [22,23]. The integration of grass clipping mulching with alfalfa planting theoretically establishes a beneficial cycle of mulch enhancing soil, improved soil fostering forage, and forage nourishing soil. Nonetheless, the underlying mechanisms—particularly the systematic effects of mulching on soil physicochemical properties, nutrient availability, and alfalfa root adaptation strategies—require rigorous investigation.

Partial least squares structural equation modeling (PLS-SEM) has emerged as a robust analytical tool for investigating complex multi-level causal relationships within agricultural and ecological systems [24]. In contrast to covariance-based SEM, PLS-SEM is particularly advantageous for small-to-medium sample sizes and prioritizes predictive accuracy, rendering it especially suitable for analyzing the cascade effects of soil amendments on plant productivity [25,26]. By decomposing the total effect of mulching into direct and indirect pathways, PLS-SEM facilitates a quantitative assessment of the relative contributions of physical, chemical, and nutrient-mediated mechanisms that influence plant performance [27,28].

Given the above considerations, this study was conducted with the following objectives: (1) to quantify the effects of different grass clipping mulching rates on soil pH, ESP, salt composition, organic matter, aggregate structure, and nutrient availability in soda saline–alkali soil; (2) to examine the dynamic responses of alfalfa emergence rate, root morphology, biomass accumulation, and photosynthetic physiology to mulching; and (3) to construct a PLS-SEM causal pathway model to elucidate the mechanistic linkages between soil amelioration and alfalfa productivity.

We hypothesized that (i) increasing mulching density would progressively improve all dimensions of soil quality, (ii) soil improvement would translate into enhanced plant physiological performance and forage yield, and (iii) the nutrient enrichment pathway would exert a stronger effect on plant response than the physical or chemical amelioration pathways. The findings are expected to provide theoretical foundations and technical references for the ecological remediation of soda saline–alkali soils and the establishment of alfalfa-based artificial grasslands.

## 2. Results

### 2.1. Effects of Grass Clipping Mulching on Soil Physical Properties

**Figure 1:**
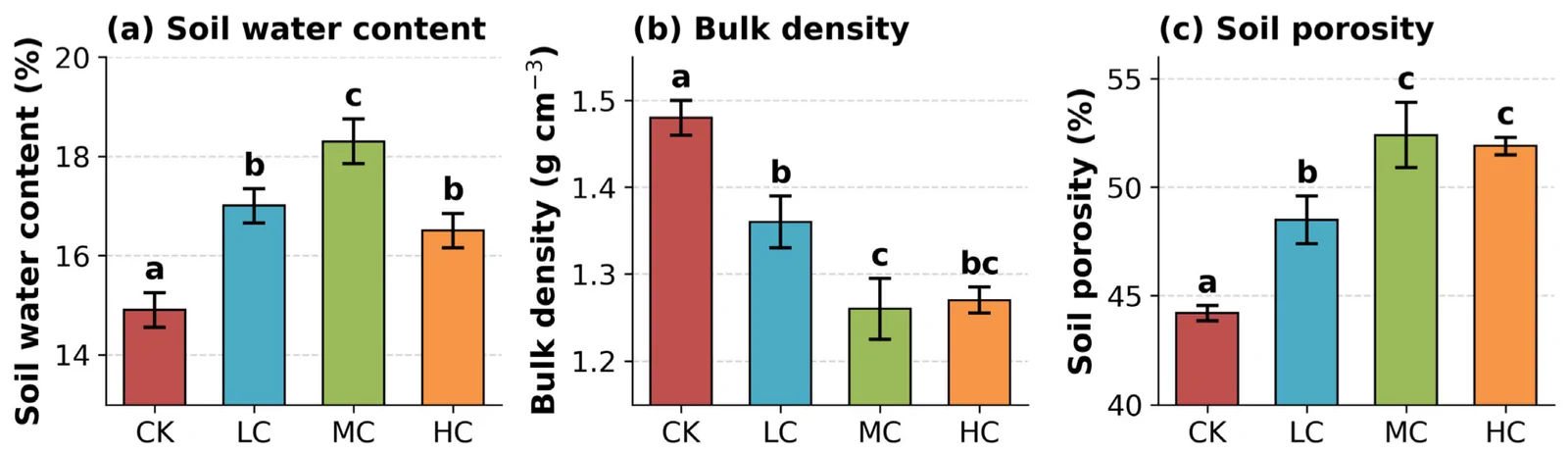
Effects of grass clipping mulching on physical properties of soda saline–alkali soil. (**a**) Soil water content; (**b**) bulk density; (**c**) soil porosity. CK, no mulching; LC, low-density grass clipping mulching; MC, medium-density grass clipping mulching; HC, high-density grass clipping mulching. Error bars indicate standard deviation (SD, *n* = 3). Different lowercase letters above bars indicate significant differences among treatments at *p* < 0.05 by Duncan’s multiple range test.

The effects of grass clipping mulching on soil physical properties were pronounced and density-dependent. As shown in Figure 1, soil water content (SWC) in the 0–20 cm layer increased significantly with mulching density, rising from 14.9% in CK to 17.0%, 18.3%, and 16.5% in LC, MC, and HC, respectively. The MC treatment exhibited the highest SWC. Soil bulk density exhibited a progressive decline with increasing mulching density, decreasing from 1.48 g cm^−3^ in CK to 1.36, 1.26, and 1.27 g cm^−3^ in LC, MC, and HC, respectively. Correspondingly, total soil porosity increased from 44.2% in CK to 48.5%, 52.4%, and 51.9% under LC, MC, and HC treatments, respectively.

### 2.2. Effects of Grass Clipping Mulching on Soil Chemical Properties

**Figure 2:**
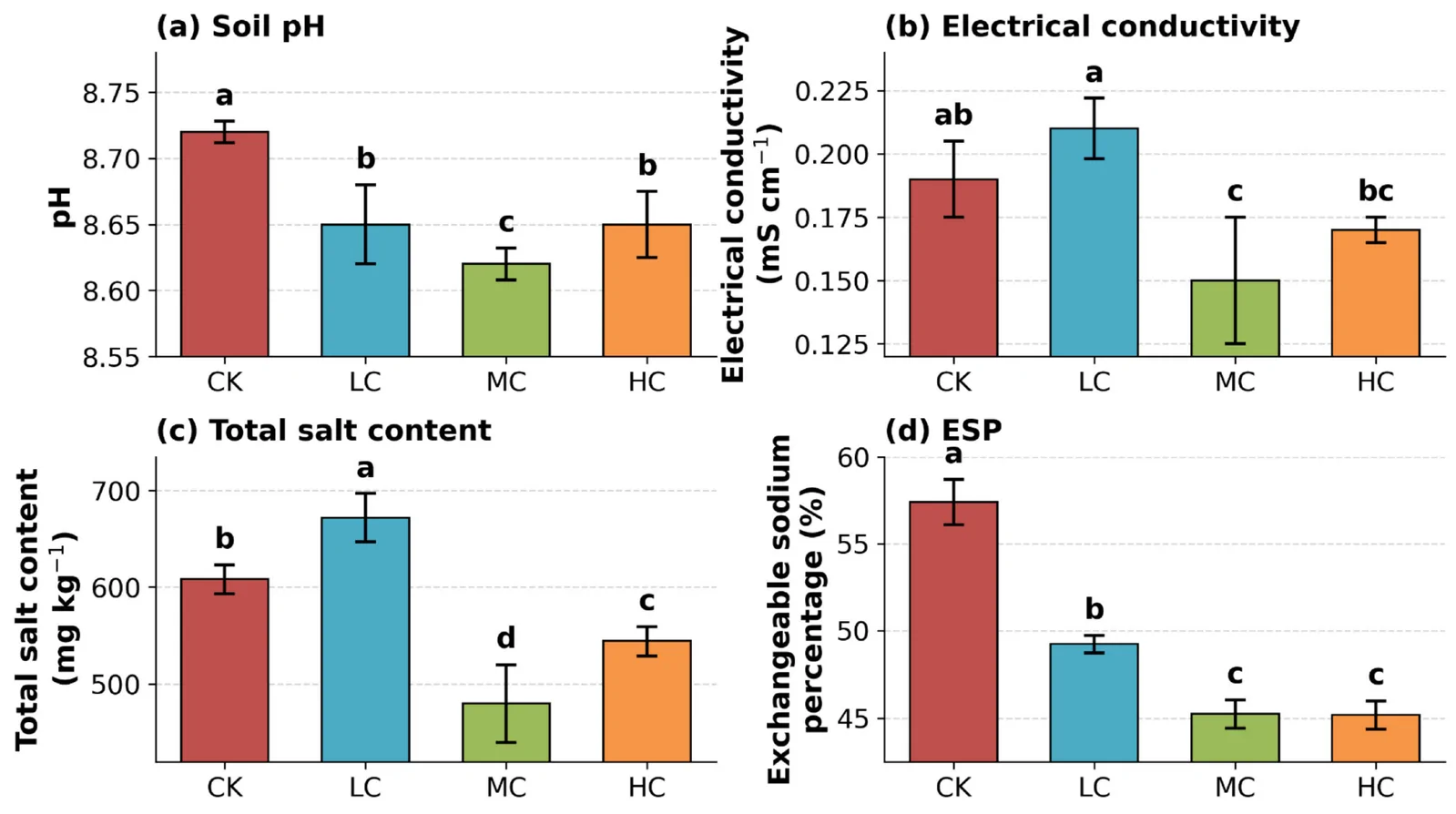
Effects of grass clipping mulching on chemical properties of soda saline–alkali soil. (**a**) Soil pH; (**b**) electrical conductivity; (**c**) total salt content; (**d**) exchangeable sodium percentage. CK, no mulching; LC, low-density mulching; MC, medium-density mulching; HC, high-density mulching. Error bars indicate standard deviation (SD, *n* = 3). Different lowercase letters above bars indicate significant differences at *p* < 0.05 by Duncan’s test.

Grass clipping mulching exerted significant ameliorative effects on the chemical properties of soda saline–alkali soil (Figure 2). Soil pH decreased progressively with increasing mulching density, from 8.72 in CK to 8.65, 8.62, and 8.65 in LC, MC, and HC, respectively. The MC treatment achieved a pH reduction of 0.10 units compared to CK (*p* < 0.01). Electrical conductivity (EC) showed an overall declining trend across mulching densities, from 0.19 mS cm^−1^ in CK to 0.15 and 0.17 mS cm^−1^ in MC and HC, respectively. Total soil salt content exhibited a similar pattern, decreasing from 608 mg kg^−1^ in CK to 480 and 544 mg kg^−1^ in MC and HC, respectively. The exchangeable sodium percentage (ESP) was significantly reduced by mulching, declining from 57.39% in CK to 49.22%, 45.24%, and 45.18% in LC, MC, and HC, respectively.

### 2.3. Effects of Grass Clipping Mulching on Soil Nutrient Properties

**Figure 3:**
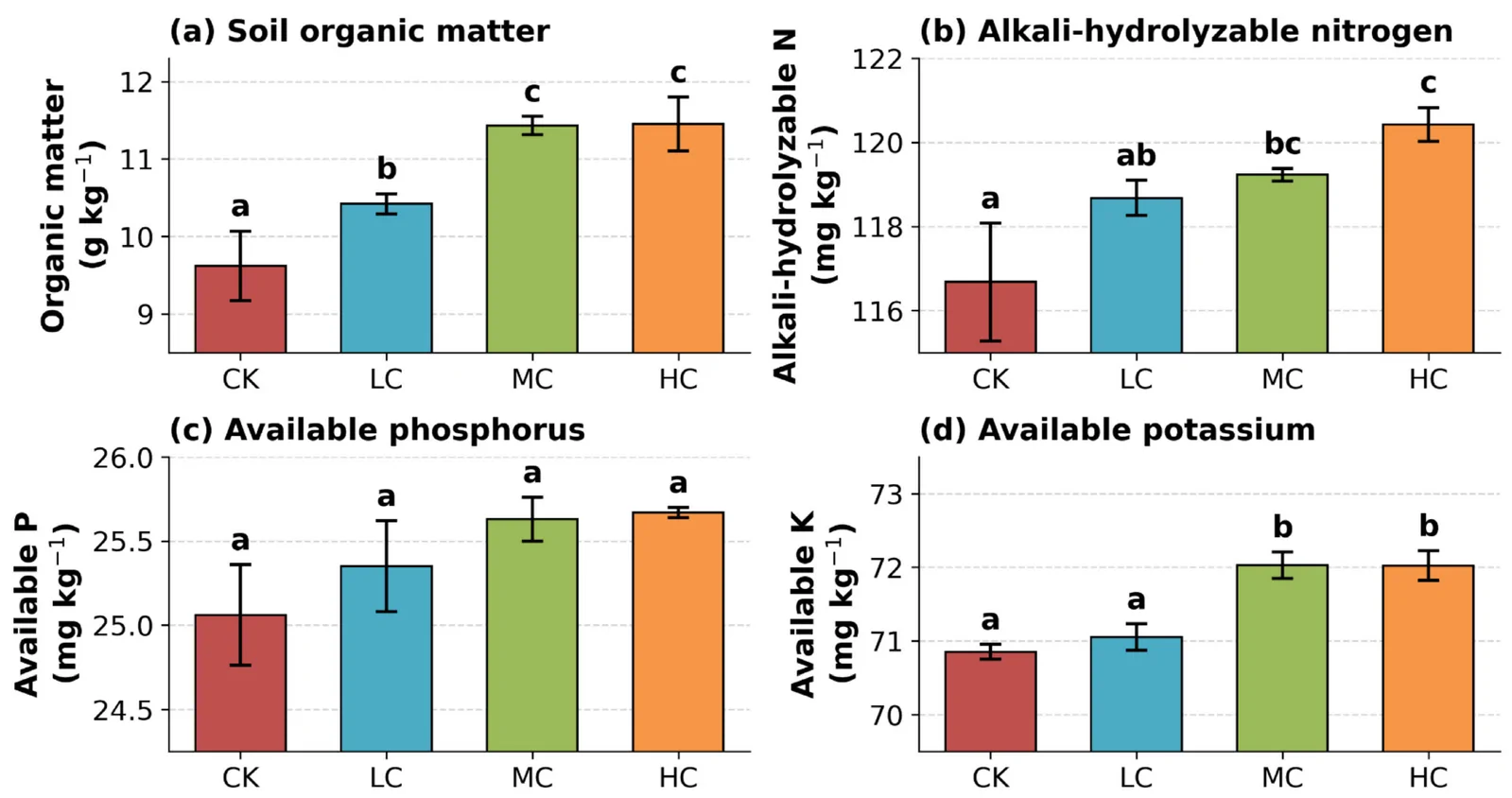
Effects of grass clipping mulching on soil nutrient properties in the second year of lucerne cultivation on soda saline–alkali soil. (**a**) Soil organic matter; (**b**) alkali-hydrolyzable nitrogen; (**c**) available phosphorus; (**d**) available potassium. CK, no mulching; LC, low-density grass clipping mulching; MC, medium-density grass clipping mulching; HC, high-density grass clipping mulching. Error bars indicate standard deviation (SD, *n* = 3). Different lowercase letters above bars indicate significant differences among treatments at *p* < 0.05 by Duncan’s multiple range test.

Organic mulching significantly enhanced soil nutrient availability. As shown in Figure 3, soil organic matter (SOM) content increased markedly with mulching density, rising from 9.6 g kg^−1^ in CK to 10.4, 11.4, and 11.5 g kg^−1^ in LC, MC, and HC, respectively. The MC and HC treatments achieved the highest SOM levels. Relative to CK (9.6 g kg^−1^), the differences for MC (11.4 g kg^−1^) and HC (11.5 g kg^−1^) were 1.8 and 1.9 g kg^−1^, equivalent to 18.8% and 19.8%, respectively. Alkali-hydrolyzable nitrogen increased from 116.7 mg kg^−1^ in CK to 118.7, 119.2, and 120.4 mg kg^−1^ in LC, MC, and HC, respectively. Available phosphorus and potassium also showed positive responses to mulching.

### 2.4. Integrated Assessment of Soil Amelioration by Grass Clipping Mulching

**Figure 4:**
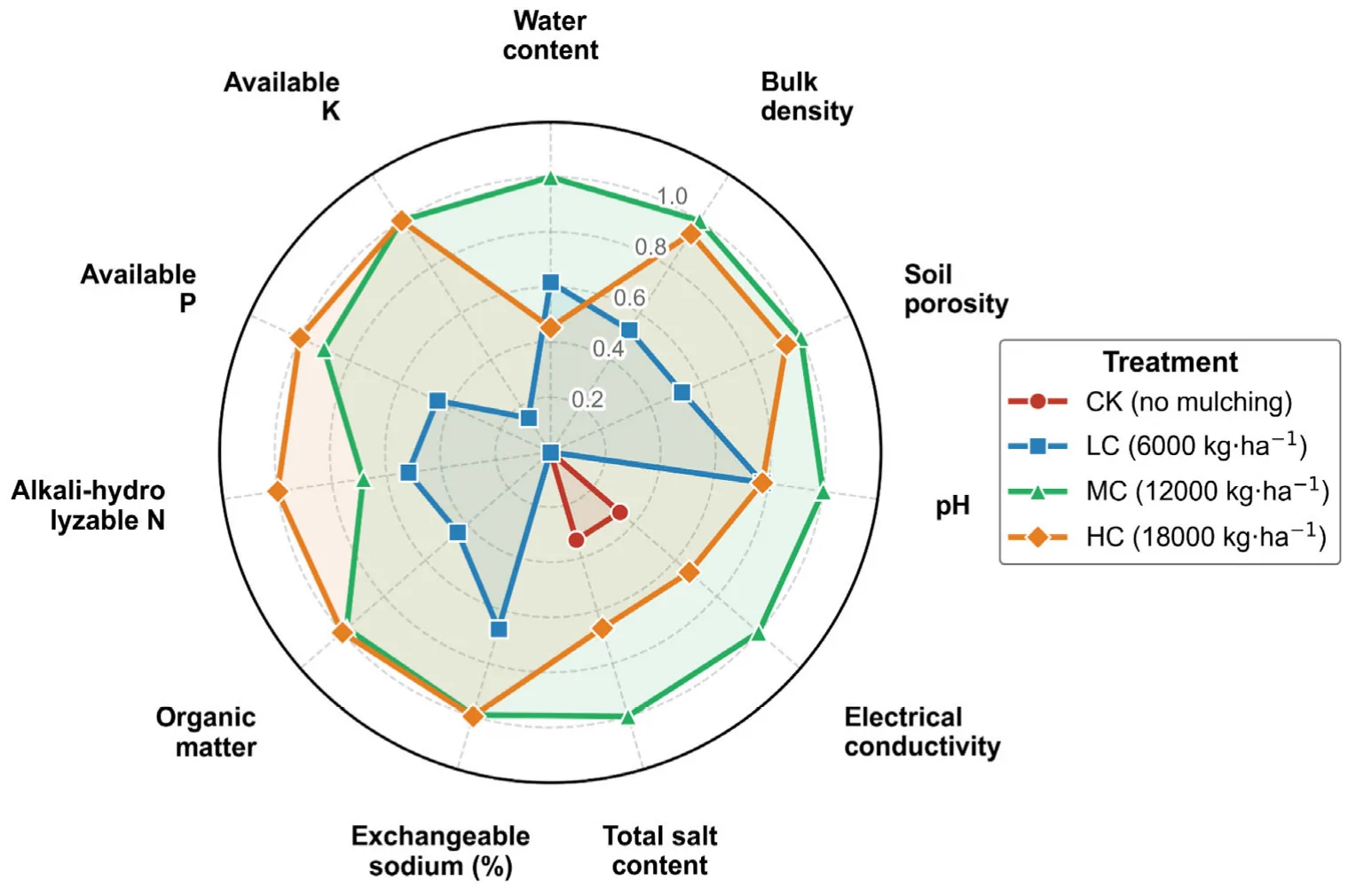
Effects of grass clipping mulching on comprehensive soil properties of soda saline–alkali soil (standardized radar chart). Note: All indicators were standardized to a 0–1 scale. Higher values indicate better amelioration. Bulk density, pH, electrical conductivity, total salt, and exchangeable sodium were reversely standardized (lower is better).

To evaluate the overall amelioration effect of grass clipping mulching on soda saline–alkali soil, a comprehensive radar chart analysis was conducted by standardizing all soil indicators to a 0–1 scale. As shown in Figure 4, the area of the radar polygon increased progressively with mulching density, indicating that the integrated soil quality improved from CK to HC. The HC and MC treatments exhibited the largest polygon areas, reflecting comprehensive improvements across physical, chemical, and nutrient dimensions. Notably, the improvements in water content, organic matter, and alkali-hydrolyzable nitrogen were the most pronounced, while bulk density, pH, electrical conductivity, total salt, and exchangeable sodium were reversed (lower values indicate better amelioration). The radar chart clearly demonstrated that grass clipping mulching achieved a multi-dimensional amelioration effect, with medium-density and high-density mulching showing the most favorable comprehensive soil quality.

### 2.5. Effects of Grass Clipping Mulching on Alfalfa Winter Survival Rate

**Figure 5:**
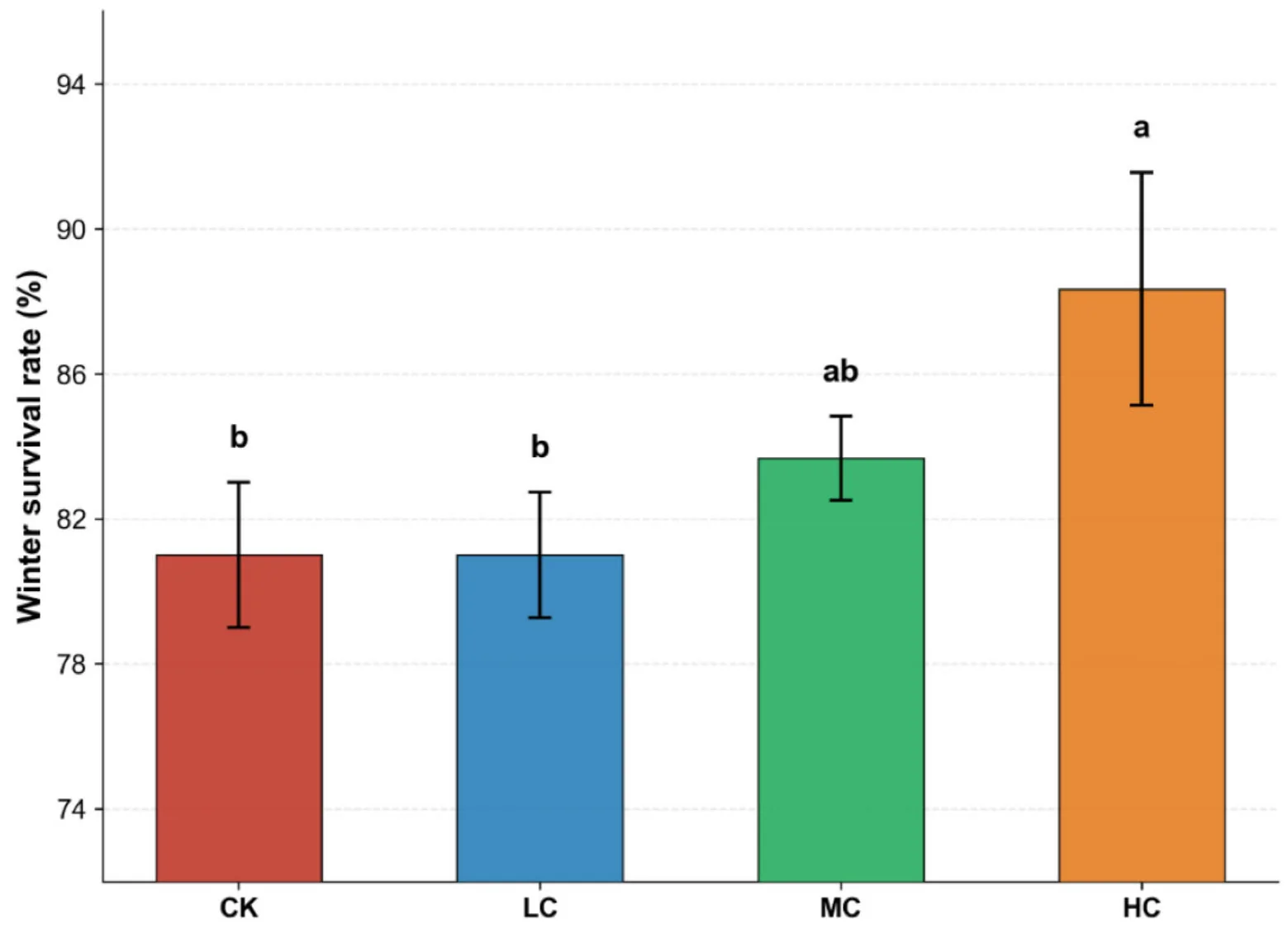
Effects of grass clipping mulching on alfalfa winter survival rate in the second year on soda saline–alkali soil. CK, no mulching; LC, low-density mulching; MC, medium-density mulching; HC, high-density mulching. Error bars indicate standard deviation (SD, *n* = 3). Different lowercase letters above bars indicate significant differences among treatments at *p* < 0.05 by Duncan’s multiple range test.

The overwintering rate of alfalfa was significantly improved by grass clipping mulching. As shown in Figure 5, the overwintering rate increased from 81.5% in CK to 81.7%, 84.8%, and 89.1% in LC, MC, and HC, respectively. The HC treatment enhanced overwintering survival by 9.3% relative to CK (*p* < 0.05). This improvement is attributed to the insulating effect of the mulch layer, which moderated soil temperature fluctuations and reduced frost damage to the root crown during winter.

### 2.6. Effects of Grass Clipping Mulching on Photosynthetic Characteristics of Alfalfa

**Figure 6:**
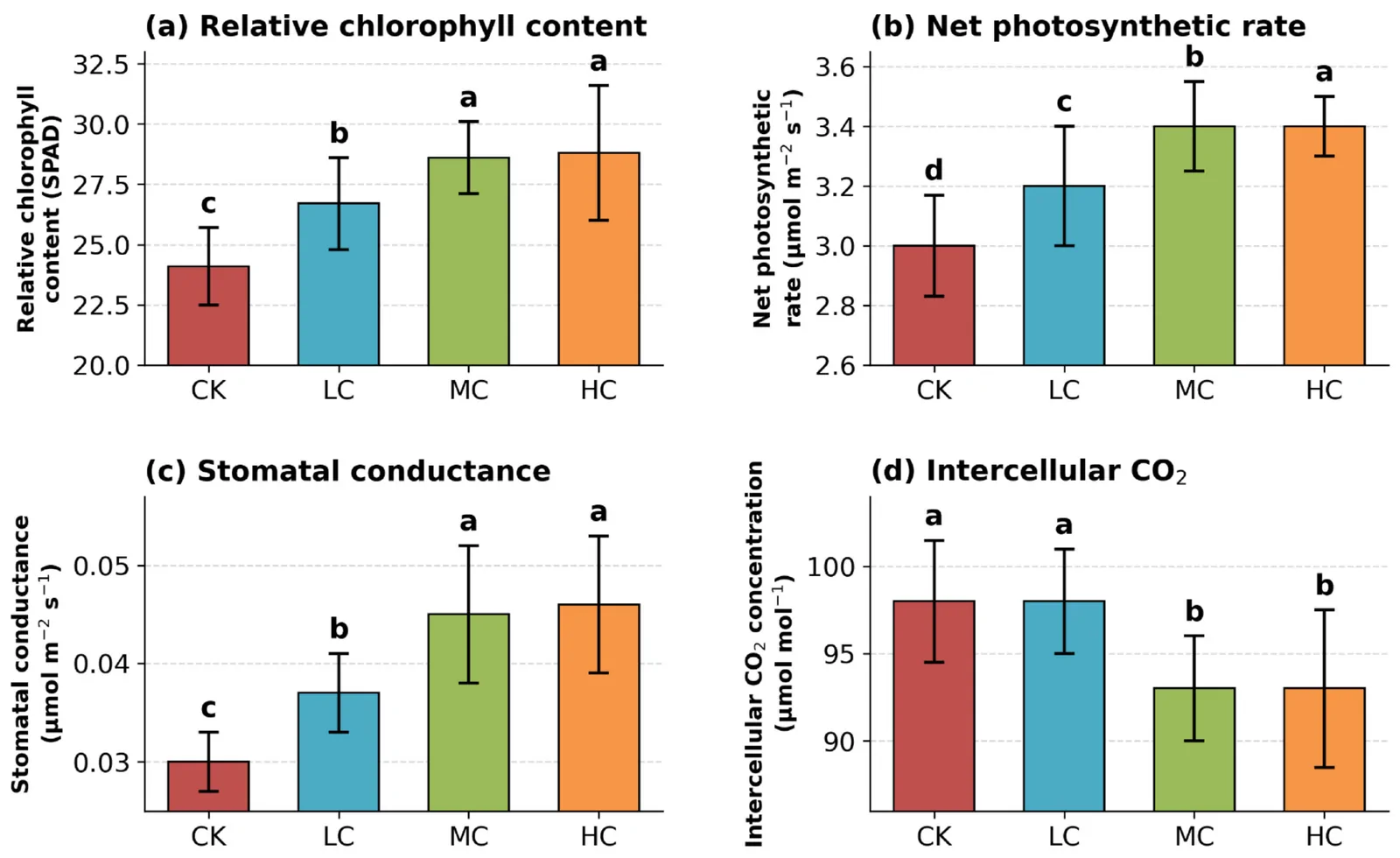
Effects of grass clipping mulching on photosynthetic characteristics of alfalfa in the second year on soda saline–alkali soil. (**a**) Relative chlorophyll content; (**b**) net photosynthetic rate; (**c**) stomatal conductance; (**d**) intercellular CO_2_ concentration. CK, no mulching; LC, low-density grass clipping mulching; MC, medium-density grass clipping mulching; HC, high-density grass clipping mulching. Error bars indicate standard deviation (SD, *n* = 10). Different lowercase letters above bars indicate significant differences among treatments at *p* < 0.05 by Duncan’s multiple range test.

As shown in Figure 6, net photosynthetic rate (Pn) increased significantly with mulching density, from 3.0 μmol m^−2^ s^−1^ in CK to 3.2, 3.4, and 3.4 μmol m^−2^ s^−1^ in LC, MC, and HC, respectively (*p* < 0.05). Stomatal conductance (Gs) followed a similar trend, increasing from 0.030 mol m^−2^ s^−1^ in CK to 0.037, 0.045, and 0.046 mol m^−2^ s^−1^ in LC, MC, and HC, respectively. Intercellular CO_2_ concentration (Ci) showed the opposite trend, decreasing from 98.0 μmol mol^−1^ in CK to 97.8, 92.3, and 92.3 μmol mol^−1^ in LC, MC, and HC, respectively. Relative chlorophyll content (SPAD) increased from 24.1 in CK to 26.7, 28.6, and 28.8 in LC, MC, and HC, respectively. The decline in Ci coupled with increased Pn and Gs under mulching treatments indicates enhanced stomatal opening and more efficient photosynthetic gas exchange.

### 2.7. Effects of Grass Clipping Mulching on Agronomic Traits and Hay Yield of Alfalfa

**Figure 7:**
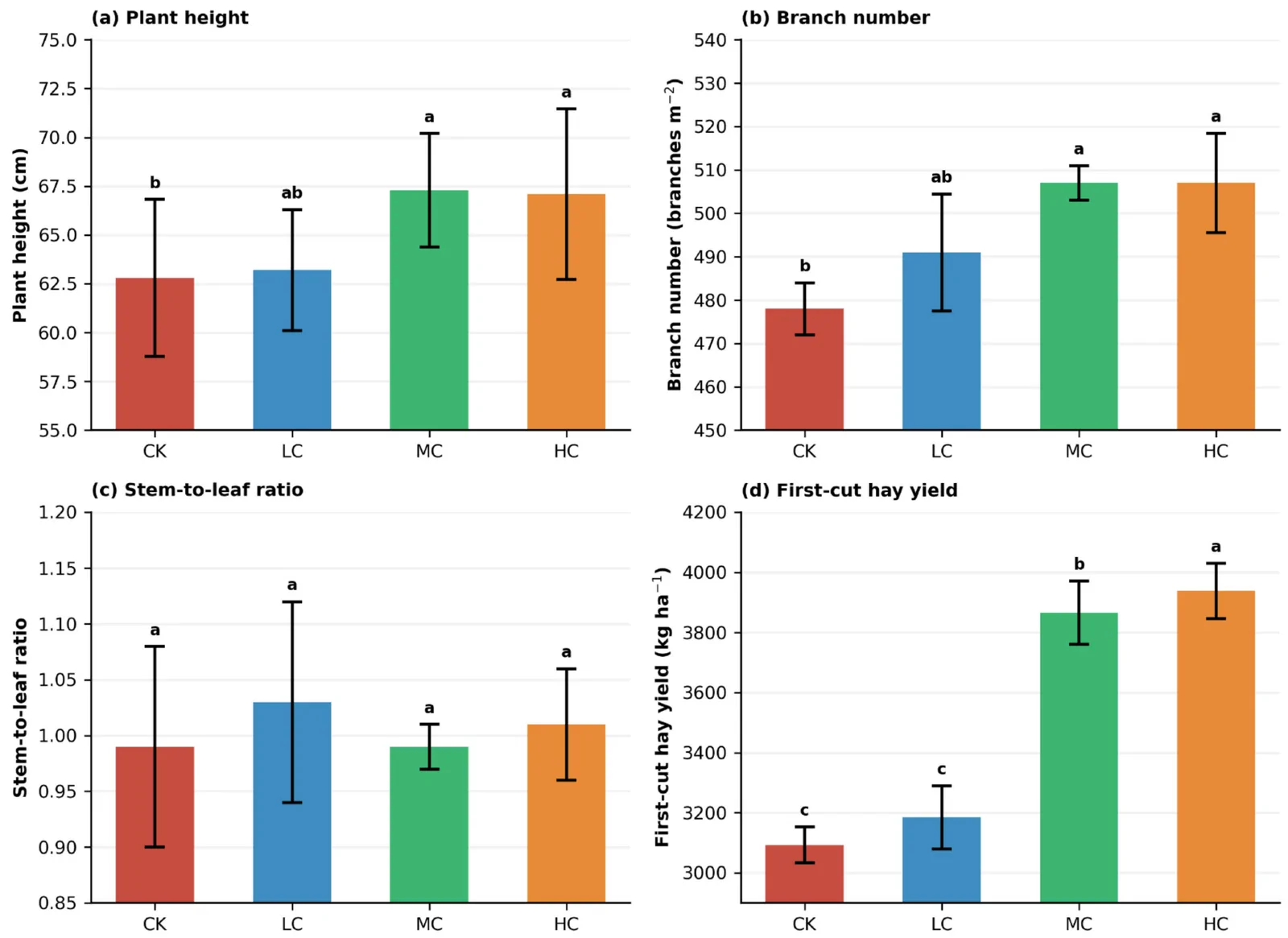
Effects of grass clipping mulching on agronomic traits and yield of alfalfa in the second year on soda saline–alkali soil. (**a**) Plant height; (**b**) branch number; (**c**) stem-to-leaf ratio; (**d**) first-cut hay yield. CK, no mulching; LC, low-density grass clipping mulching; MC, medium-density grass clipping mulching; HC, high-density grass clipping mulching. Error bars indicate standard deviation (SD). Different lowercase letters above bars indicate significant differences among treatments at *p* < 0.05 by Duncan’s multiple range test.

At the initial flowering stage (15 June 2024), plant height increased significantly with mulching density, from 62.8 cm in CK to 63.2, 67.3, and 67.1 cm in LC, MC, and HC, respectively (Figure 7). Branch number per square meter also increased markedly, from 478 branches m^−2^ in CK to 491, 507, and 507 branches m^−2^ in LC, MC, and HC, respectively. The stem-to-leaf ratio remained relatively stable across all treatments, ranging from 0.99 to 1.03, with no significant differences between the control and mulching treatments (*p* > 0.05), indicating that mulching density had no notable effect on the allocation ratio between stem and leaf biomass. First-cut hay yield increased significantly from 3093 kg ha^−1^ in CK to 3185, 3866, and 3938 kg ha^−1^ in LC, MC, and HC, respectively.

### 2.8. Effects of Grass Clipping Mulching on Forage Quality of Alfalfa

To evaluate whether the documented yield improvements were accompanied by nutritional quality enhancement, forage quality indicators were analyzed in the first-cut harvest (Table 1). Grass clipping mulching significantly improved the nutritional quality of alfalfa hay. Crude protein (CP) content increased progressively with mulching density, from 18.87% in CK to 20.42% in HC, representing an 8.2% improvement (*p* < 0.05). MC achieved an intermediate value of 19.84%, sharing the letter “a” with HC (20.42%) in Duncan’s grouping, which confirms that MC and HC were not significantly different from each other, while both were significantly higher than CK (18.87%) and LC (19.47%). Crude fat (CF) content also responded positively, with MC (3.59%) and HC (3.53%) both significantly exceeding CK (3.12%) and LC (3.21%), with increases of 15.1% and 13.1%, respectively.

Acid detergent fiber (ADF), which primarily reflects lignin and cellulose content, decreased significantly under MC (29.21%) and HC (28.20%), compared with CK (31.72%) and LC (30.75%). The 11.1% reduction in ADF under HC indicated decreased lignification and improved fiber digestibility. In contrast, neutral detergent fiber (NDF) showed no significant differences among treatments (*p* > 0.05), ranging narrowly from 37.27% (HC) to 37.69% (CK). This differential response suggested that mulching selectively reduced lignin and cellulose fractions without affecting total cell wall content.

Relative feed value (RFV), the integrated index of forage quality, increased progressively from 158.43 (CK) to 167.08 (HC). MC and HC achieved 3.8% and 5.5% improvements over CK, respectively, with all treatments exceeding the premium quality threshold (RFV > 150) for dairy cattle. The resulting decrease in the ADF/NDF ratio under HC (0.76 vs. 0.84 in CK) confirmed a shift toward more digestible fiber architecture.

### 2.9. Structural Equation Modeling of the Causal Pathways from Mulching to Forage Production

**Figure 8:**
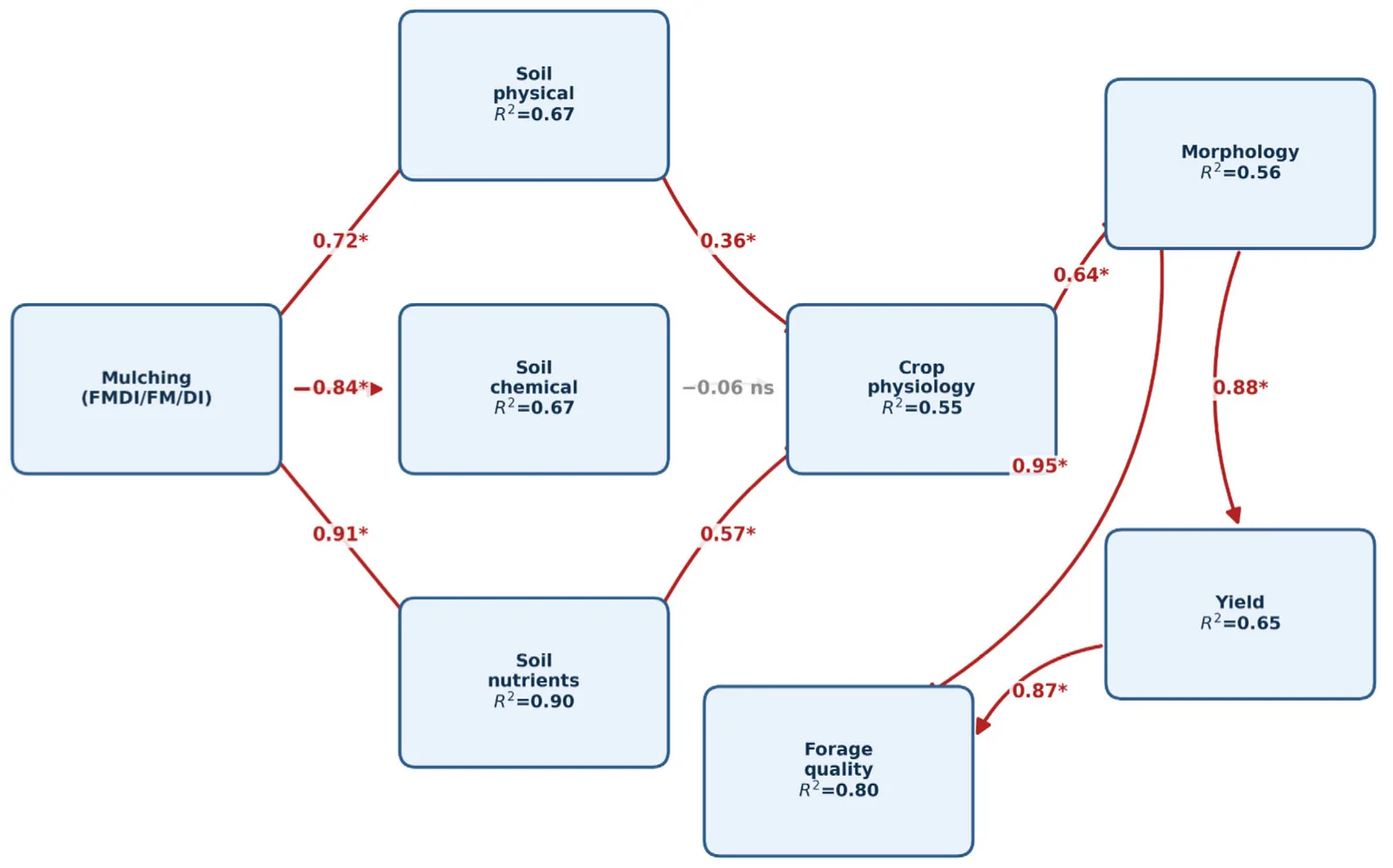
Conceptual structural equation model showing the causal pathways from grass clipping mulching to forage yield and quality in soda saline–alkali soil. Standardized path coefficients are shown on arrows, with significance indicated by * *p* < 0.05. R^2^ values indicate the proportion of variance explained in each endogenous latent variable. Model fit: GoF = 0.76 (strong), SRMR = 0.07, Bootstrap = 5000 resamples, ns = not significant.

Partial least squares structural equation modeling (PLS-SEM) was employed to quantify the multi-level causal pathways linking soil amelioration to alfalfa yield and quality. As shown in Figure 8, the structural model revealed that grass clipping mulching density exerted the strongest direct effect on soil nutrient enrichment (standardized path coefficient β = 0.91, *p* < 0.001), followed by soil chemical amelioration (β = 0.84, *p* < 0.001) and soil physical improvement (β = 0.72, *p* < 0.001). Soil nutrient enrichment had the strongest direct effect on plant physiological enhancement (β = 0.57, *p* < 0.001), whereas soil physical improvement showed a moderate direct effect (β = 0.36, *p* < 0.01). Notably, the direct path from soil chemical amelioration to plant physiological enhancement was non-significant (β = −0.06, *p* > 0.05). The measurement model was assessed prior to structural path interpretation. All standardized outer loadings exceeded the recommended threshold of 0.70 (range: 0.71–0.93), confirming adequate indicator reliability. Internal consistency reliability was supported by Cronbach’s alpha (α = 0.78–0.91) and composite reliability (CR = 0.85–0.94) for all latent constructs, both above the conventional benchmark of 0.70. Convergent validity was established as the average variance extracted (AVE) and ranged from 0.58 to 0.76, exceeding the 0.50 criterion. Discriminant validity was confirmed by the heterotrait–monotrait ratio of correlations (HTMT), with all values below the conservative threshold of 0.85. Model fit was additionally assessed using the standardized root mean square residual (SRMR = 0.07), which is below the 0.08 threshold, indicating acceptable model fit. Bootstrap confidence intervals (5000 resamples) for all significant path coefficients did not include zero, confirming path stability.

Plant physiological enhancement significantly promoted plant morphological development (β = 0.64, *p* < 0.001), which in turn enhanced forage yield production (β = 0.88, *p* < 0.001) and forage quality improvement (β = 0.95, *p* < 0.001). Forage yield production also positively influenced forage quality improvement (β = 0.87, *p* < 0.001). The coefficients of determination (R^2^) for the endogenous latent variables were 0.67 for soil physical improvement, 0.67 for soil chemical amelioration, 0.90 for soil nutrient enrichment, 0.80 for plant physiological enhancement, 0.55 for plant morphological development, 0.56 for forage yield production, and 0.65 for forage quality improvement. The global goodness-of-fit (GoF) index for the model was 0.76, indicating a substantial predictive relevance of the structural model. Because the dataset comprised 12 plots (4 treatments × 3 replicates), we acknowledge that the modest sample size may affect the precision of path coefficient estimates; accordingly, all inferential conclusions were based on bias-corrected bootstrap confidence intervals rather than parametric *p*-values alone. Potential common method bias was examined using the full collinearity test; the variance inflation factors (VIFs) for all constructs were below the conservative threshold of 3.3, suggesting that common method bias is unlikely to substantially bias the structural estimates.

## 3. Discussion

### 3.1. Soil Amelioration as the Foundation of Mulching Effects

The ameliorative effect of grass clipping mulching on soda saline–alkali soils operates not through isolated pathways but via a cascading feedback loop involving physical restructuring, chemical conditioning, and biological activation, culminating in a comprehensive soil remediation outcome. The mulch layer acts as a physical barrier that curtails evaporation and enhances infiltration; its decomposition products participate in ion exchange and acid-base neutralization, and the concomitant stimulation of microbial mineralization collectively synchronizes the optimization of soil water–salt balance, pH buffering, and nutrient supply [29].

Grass powder mulching markedly improved soil moisture status and structural stability. By intercepting solar radiation and interrupting the soil–atmosphere interface, the mulch layer suppressed the first (constant-rate) stage of soil evaporation and reduced evaporative water loss, leading to a significant increase in soil water content (SWC) under MC relative to CK (18.3% vs. 14.9%). Concurrently, organic matter released during the gradual decomposition of the surface-applied grass powder fostered aggregate formation and pore development, reducing bulk density and increasing total porosity. This structural reorganization not only enhanced water-holding and conducting capacity but also provided preferential flow pathways for salt leaching [30]. Similar findings have been reported for organic mulching: grass-clipping mulch increased saturated water content by 8–12% in low-permeability soils [31], and straw or grass mulching has been shown to improve infiltration and water retention in saline soils [29]. However, the physical benefits exhibited a density-dependent threshold: medium-density application outperformed the high-density treatment in soil water content (18.3% vs. 16.5%) and total porosity (52.4% vs. 51.9%). We attribute this slight decline under high-density mulching to the excessively thick mulch layer impeding soil–atmosphere gas exchange and attenuating soil temperature, which may transiently slow microbial decomposition and root respiration, thereby compromising water infiltration and aeration relative to the optimal MC treatment [32,33]. This nonlinear response underscores the need to balance water conservation against soil aeration when determining optimal mulching rates [34]. The magnitude of the physical improvements observed here—the 22.8% increase in soil water content and the 18.6% increase in total porosity under MC relative to CK—is consistent with, or exceeds, the values reported for organic mulching [29,31].

The improvement in soil chemical properties is a direct extension of physical optimization. Organic acids and CO_2_ released during grass clipping decomposition neutralize soil carbonates, significantly reducing pH [17]; concurrently, Ca^2+^ and Mg^2+^ liberated through mineralization displace exchangeable Na^+^ from soil colloids [30], decreasing the exchangeable sodium percentage (ESP) from 57.4% to 45.2%, a critical metric in soda saline–alkali soils, where high ESP induces clay dispersion and structural degradation [35]. Studies have demonstrated that organic amendments can synergistically lower both pH and ESP while raising cation exchange capacity [36]. The decline in ESP, together with enhanced permeability driven by improved infiltration, facilitates the leaching of total soluble salts from the surface layer [37]. Zhou et al. found that the application of acidic corn stalk biochar (ACSBC) markedly enhanced the chemical properties of alkaline saline–alkali soils: soil water, nutrient elements, cation exchange capacity, and organic matter increased, while the soil pH decreased by an average of 0.3 units, salinity declined by 19.37%, and exchangeable sodium percentage (ESP) declined [38], closely aligning with our findings. The dose-dependent decrease in exchangeable Na^+^ content can be attributed to the progressively greater release of Ca^2+^, Mg^2+^ and organic acids from larger amounts of decomposing mulch [39,40]; the divalent cations preferentially occupy the exchange sites of soil colloids and displace Na^+^ into the soil solution [41], while the improved infiltration and reduced ESP facilitate the downward leaching of Na^+^ out of the root zone [42]; the simultaneous decline in pH further increases the negative surface charge of soil colloids, enhancing Na^+^ displacement [43]. The 0.10-unit pH decrease observed under MC is smaller than the 0.3-unit reduction reported by Zhou et al. for acidic biochar amendment [38], which is expected given the weaker acidity of decomposing grass residues; by contrast, the 21.2% reduction in ESP achieved here with a low-cost residue is comparable to the improvements typically obtained with gypsum-based amendments [10].

Notably, the transient increase in EC and total salt content under low-density mulching (LC) relative to CK can be attributed to the insufficient mulch thickness to fully suppress soil evaporation; residual capillary rise may have transported dissolved salts from deeper layers to the surface during the early growing season, a phenomenon consistent with short-term salt redistribution under incomplete surface cover [44]. Under medium- and high-density mulching, the thicker and more continuous mulch layer effectively interrupted the evaporative flux, thereby promoting salt leaching and reducing surface accumulation [45].

The enhancement of nutrient availability is predicated on the reactivation of microbial activity, which in turn depends on the ameliorated physicochemical environment [46,47]. Grass clippings directly contribute organic matter to the soil carbon pool [48]; more importantly, the alleviation of saline–alkali stress relieves the suppression of key enzymes such as urease and phosphatase, allowing microbial communities to restore their mineralization functions [49,50]. This interpretation is consistent with the findings of Song et al., who reported that straw mulching in saline–alkali land in North China could generally increase soil organic matter by 19% and reduce salt content by 15% [51]. Consequently, the contents of alkali-hydrolysable nitrogen, available phosphorus, and available potassium were significantly elevated. This increase is not merely a result of exogenous nutrient input but rather a product of the cascading sequence “chemical desalinization—microbial reactivation—nutrient release” [52,53]. The centrality of this soil-mediated pathway is further supported by Liao et al., who showed that the effect of straw mulching on wheat yield is transmitted through the interactions among soil organic carbon, available nitrogen, phosphorus and potassium, bulk density, and soil moisture [54]—a causal architecture that mirrors the PLS-SEM pathways quantified in Section 3.3 of this study. Similarly, Wang et al. demonstrated that organic amendment-driven enhancement of soil organic matter and available nitrogen underlies the growth response of maize in saline–alkali soil [55].

The approximately 19–20% higher SOM concentrations in MC and HC should be interpreted cautiously. The absolute differences from CK were 1.8 and 1.9 g kg^−1^, and the relatively low CK value (9.6 g kg^−1^) makes these differences appear large as percentages. Soil was sampled approximately eight months after mulch application; this comparison at one date is not a measured change from pretreatment values. Mulch-derived fragments or root material may have contributed to the measured SOM, although their contributions were not quantified. Because bulk density also differed among treatments, concentration differences do not directly establish changes in soil organic matter stocks or long-term carbon sequestration. Repeated sampling and stock measurements on an equivalent-soil-mass basis are needed to determine whether the response persists.

Collectively, the dose-dependent improvement of soil quality by grass clipping mulching can be explained by a coherent set of mechanisms that operate simultaneously. First, increasing mulch density strengthens the surface barrier, further suppressing evaporation and maintaining higher soil water content. Second, the greater input of decomposable organic matter supplies more polysaccharides and humic substances that cement soil particles into stable aggregates, thereby lowering bulk density and increasing porosity. Third, larger amounts of organic acids and CO_2_ released during decomposition more effectively neutralize soil alkalinity, lowering pH. Fourth, the enhanced release of Ca^2+^ and Mg^2+^, combined with the improved leaching conditions created by better infiltration and lower ESP, displaces exchangeable Na^+^ from the soil colloid complex and promotes its removal from the root zone, so that EC and total salt content decline. Finally, the larger carbon and nutrient input, together with the relieved saline–alkali stress. These mechanisms explain why the integrated soil quality, as shown by the radar chart analysis, improved progressively from CK to HC.

### 3.2. Translating Soil Amelioration to Enhanced Plant Performance

The improved soil physical, chemical, and nutrient conditions collectively translated into enhanced alfalfa performance across multiple dimensions—photosynthetic efficiency, overwintering survival, agronomic traits, and forage yield and quality. The increased net photosynthetic rate (Pn) from 3.0 to 3.4 μmol m^−2^ s^−1^ under mulching, coupled with increased stomatal conductance (Gs) and decreased intercellular CO_2_ concentration (Ci), indicates that the enhancement of photosynthesis was primarily driven by stomatal factors rather than non-stomatal limitations. The improved soil water availability and reduced salt stress under mulching likely alleviated stomatal closure induced by osmotic stress, allowing greater CO_2_ influx and photosynthetic carbon assimilation [56,57]. The increase in relative chlorophyll content (SPAD) reflects improved chlorophyll synthesis and reduced chlorophyll degradation [58]. Salt stress is known to cause chlorophyll degradation and disrupt photosystem II (PSII) structure, leading to impaired photosynthetic electron transport [59,60]. This interpretation is consistent with studies demonstrating that improved soil conditions enhance PSII photochemical efficiency and electron transport capacity in alfalfa under abiotic stress [61].

The improvement in overwintering rate from 81.5% in CK to 89.1% in HC is particularly noteworthy for alfalfa production in Northeast China, where winter injury is a major constraint. The mechanisms likely involve multiple protective effects: the mulch layer provides thermal insulation that reduces soil temperature fluctuations and protects root crowns from lethal freezing [62]; improved soil structure and drainage reduce ice lens formation that can physically damage roots; and enhanced carbohydrate reserves resulting from better growing-season photosynthesis provide greater cryoprotective capacity [63]. A study analyzing the rhizosphere microenvironment of alfalfa showed that soil amendments enhance alfalfa’s cold tolerance by increasing the content and availability of soil nutrients, including total carbon (TC), total nitrogen (TN), total phosphorus (TP), and total potassium (TK) [64]. Collectively, grass mulch enhances alfalfa winter hardiness through synergistic effects of thermal protection, reserve accumulation, mechanical stress reduction, and nutrient supply.

First-cut hay yield increased by 27.3% under HC, demonstrating the substantial productivity gains achievable through soil amelioration. The yield response was density-dependent, with MC and HC achieving comparable yields, suggesting an optimal application rate between 12,000 and 18,000 kg ha^−1^. The yield improvement was accompanied by enhanced forage quality. The SEM cascade provides a quantitative explanation for these quality gains: mulching density drove soil nutrient enrichment (β = 0.91), which enhanced plant physiology (β = 0.57), promoted morphological development (β = 0.64), and ultimately improved forage quality (β = 0.95). The increase in crude protein reflects both enhanced nitrogen availability via the nutrient enrichment pathway and improved plant nitrogen metabolism under reduced salt stress [65,66]. The increase in crude fat further contributed to improved energy density. The reduction in acid detergent fiber (ADF) indicates decreased lignification and improved cell wall digestibility [67,68]. The differential response of ADF versus neutral detergent fiber (NDF), which showed no significant change, suggests that mulching selectively reduced lignin and cellulose fractions rather than total cell wall content. This selective effect may reflect altered partitioning of photosynthetic assimilates under improved growing conditions, with more resources allocated to protein synthesis and less to structural carbohydrates [69]. RFV is an integrated index that combines digestible dry matter intake and digestible energy, reflecting both forage intake potential and nutritive value [70]. The resulting increase in relative feed value (RFV) from 158.43 to 167.08 confirms that the soil amelioration translated into tangible improvements in the feeding value of alfalfa hay. The decrease in the ADF/NDF ratio under HC further confirms a shift toward more digestible fiber architecture. These quality improvements have direct economic implications, as premium-quality alfalfa hay commands higher market prices and supports superior animal performance in dairy production systems.

### 3.3. Causal Pathway Quantification: Insights from Structural Equation Modeling

The PLS-SEM analysis provided quantitative insights into the causal architecture linking mulching to forage production, revealing that the effects are predominantly indirect and mediated through soil amelioration. The model explained substantial proportions of variance in endogenous variables (R^2^ values ranging from 0.55 to 0.90), with a global goodness-of-fit index of 0.76 indicating strong predictive relevance.

The finding that mulching density exerted the strongest direct effect on soil nutrient enrichment (β = 0.91), followed by soil chemical amelioration (β = 0.84) and soil physical improvement (β = 0.72), aligns with the mechanistic understanding that organic matter input is the primary driver of soil quality improvement in degraded soils [71]. The direct effect on nutrient enrichment exceeding that on physical properties reflects the immediate contribution of grass clippings to the soil organic matter pool, whereas physical improvements require more time for aggregate formation and structural reorganization [72,73].

The non-significant direct path from soil chemical amelioration to plant physiological enhancement (β = −0.06, *p* > 0.05) is particularly instructive. Ecologically, this pattern implies that chemical desalination and alkalinity reduction are necessary but not sufficient conditions for plant response: the lowered pH (8.72 to 8.62) and ESP (57.4% to 45.2%) create a less hostile root environment, yet the plant can only capitalize on this improvement when nutrient supply (β = 0.57) and root-zone physical conditions (β = 0.36) are simultaneously optimized. In other words, chemical amelioration functions as an enabling precondition that must be translated through physical structure and nutrient cycling before physiological benefits are realized. This hierarchical pathway underscores the importance of integrated soil management: addressing soil chemistry alone, without concomitant improvements in physical structure and nutrient status, may yield limited plant responses. The strongest driver of plant physiological enhancement was soil nutrient enrichment (β = 0.57), followed by soil physical improvement (β = 0.36). This is consistent with the understanding that nutrient availability and root-zone physical conditions (water, aeration) are the most proximate determinants of plant physiological function [74]. Plant physiological enhancement then strongly promoted morphological development (β = 0.64), which in turn drove both yield production (β = 0.88) and quality improvement (β = 0.95). The strong positive path from yield production to quality improvement (β = 0.87) indicates that the enhanced biomass production under mulching did not come at the expense of quality—rather, yield and quality improvements were synergistic. The high coefficients of determination (R^2^ = 0.80 for plant physiological enhancement, R^2^ = 0.90 for soil nutrient enrichment) and the substantial global goodness-of-fit (GoF = 0.76) confirm that the structural model effectively captured the complex interactions within the soil–plant system. These findings align with recent applications of SEM in forage production systems, where multi-level pathway analysis has been used to disentangle the trade-offs and feedbacks among yield, nutritional quality, and soil functionality [75].

### 3.4. Implications for Sustainable Alfalfa Production in Saline–Alkali Regions

The findings of this study have important implications for sustainable forage production in soda saline–alkali regions of Northeast China and similar environments worldwide. Grass clipping mulching offers a practical, cost-effective, and environmentally sustainable alternative to conventional amendments such as gypsum or chemical fertilizers for saline–alkali soil reclamation [36]. The density-dependent responses observed in this study provide practical guidance for application rates. The optimal mulching density appears to be in the medium-to-high range (1.2–1.8 kg m^−2^; 12,000–18,000 kg ha^−1^; 12–18 t ha^−1^), where the marginal benefits of additional mulching begin to plateau while the costs of material and application continue to increase. Our results show that the high-density treatment (1.8 kg m^−2^) achieved slightly higher absolute yields and quality than the medium-density treatment (1.2 kg m^−2^), but the incremental gain may not justify the additional material cost in all economic contexts. A cost–benefit analysis, incorporating local grass clipping prices, labor costs, and forage market values, would be necessary to determine the economically optimal application rate for specific farm conditions.

In addition, the use of mixed herbaceous grass as a mulching material offers additional sustainability benefits. It provides a value-added utilization pathway for agricultural residues that are otherwise often burned or discarded, contributing to air pollution and resource waste. The incorporation of grass clippings into the soil carbon pool also enhances long-term carbon sequestration, contributing to climate change mitigation goals while simultaneously improving soil health and agricultural productivity [75].

### 3.5. Limitations and Future Directions

Several limitations of this study should be acknowledged: (1) Although the experiment spanned two growing seasons, soil and plant measurements were collected at a single time point (15 June 2024, initial flowering stage); consequently, the temporal trajectory of changes in soil properties following mulching application could not be captured. Future studies incorporating seasonal or continuous monitoring are needed to characterize the time course of amelioration. (2) Soil microbial communities and enzyme activities—the likely proximate drivers of the nutrient enrichment pathway—were not measured; future work employing metagenomic, metabolomic, or enzyme-activity profiling could elucidate the microbial mechanisms underlying organic matter decomposition and nutrient mineralization. (3) Only one alfalfa cultivar (Stockpile) was evaluated; cultivar-specific differences in salt tolerance, root architecture, and nutrient use efficiency may limit the generalizability of the results, and multi-cultivar validation is warranted. (4) The two-year duration, while sufficient to detect significant treatment effects, may not capture long-term dynamics of soil organic matter accumulation and structural stabilization. (5) The potential for nitrogen immobilization during decomposition of high-carbon grass residues was not addressed and warrants investigation, particularly in the context of nitrogen management for subsequent crops.

## 4. Materials and Methods

### 4.1. Experimental Site

**Figure 9:**
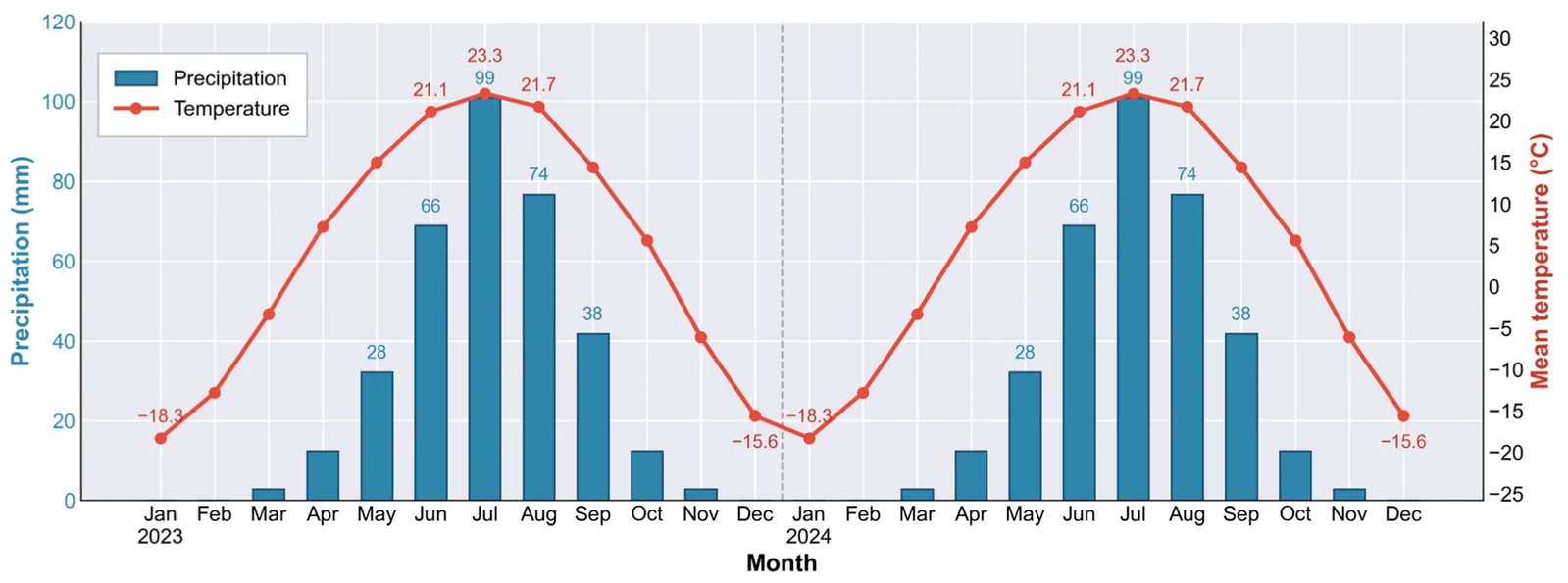
Monthly mean temperature and precipitation in Daqing (2023–2024).

The experiment was conducted at the Yinlang Ranch, Ranghulu District, Daqing City, Heilongjiang Province, China (46°27′20″ N, 124°44′70″ E, altitude 151.5 m). The region is characterized by a mid-temperate continental monsoon climate, with an average annual temperature of 5.2 °C, a frost-free period of 143 days, a mean annual wind speed of 3.9 m s^−1^, and an annual sunshine duration of 2726 h. The monthly precipitation distribution during the experimental period (2023–2024) is shown in Figure 9. The experimental field was previously cultivated with forage oats (*Avena sativa* L.) and exhibited moderate salinization. Naturally occurring halophytic vegetation, including *Chloris virgata*, *Leymus chinensis*, and *Puccinellia distans*, was sporadically observed in the surrounding area. The basic physicochemical properties of the experimental soil are presented in Table 2. No chemical fertilizer had been applied before the experiment, and none was applied during the experimental period.

### 4.2. Experimental Design

The alfalfa (*Medicago sativa* L.) cultivar used in this study was Stockpile (provided by Dairyland Seed Co., Inc., West Bend, WI, USA). Seeds were sown in July 2023 at a rate of 22.5 kg ha^−1^, with a row spacing of 12 cm. A single-factor randomized block design was adopted, with four mulch treatments based on grass clipping application rate (Table 3). Each treatment consisted of three replicates, resulting in a total of 12 plots. Each plot measured 20 m^2^ (4 m × 5 m). On 9 October 2023, grass clippings were applied to the LC, MC, and HC plots at rates of 6000, 12,000, and 18,000 kg ha^−1^ (6.0, 12.0, and 18.0 t ha^−1^), respectively; the CK plot received no mulch. All rates refer to as-applied air-dry mass. The material was manually spread evenly over the soil surface between alfalfa rows. The mulch material was sourced from moldy, deteriorated hay clippings at the Red Grassland Ranch, Daqing City, with *Leymus chinensis* as the primary component, and the organic matter, total nitrogen, total phosphorus, and total potassium contents on a dry-matter basis were 18.12%, 0.41%, 0.15%, and 0.15%, respectively. Unified field management practices were implemented across all plots during the experimental period.

### 4.3. Measurement and Analysis

#### 4.3.1. Soil Physicochemical Properties

Soil samples were collected on 15 June 2024, coinciding with the alfalfa yield measurement, from the 0–20 cm soil layer. No effective rainfall occurred within 72 h prior to sampling. To ensure representativeness, the soil of each plot was sampled at five points distributed in an S-shaped pattern and mixed into one composite sample after removal of visible plant residues and roots.

(1) Soil water content, bulk density, and porosity

Soil water content was determined by the oven-drying method. Bulk density was measured using the core ring method. Soil porosity was calculated from bulk density and particle density (2.65 g cm^−3^).

(2) Soil pH and electrical conductivity

Ten grams of prepared soil sample were shaken with distilled water at a soil-to-water ratio of 1:5, allowed to stand, and filtered. Soil pH was measured using a PHSJ-4A pH meter (Leici, Shanghai, China), and electrical conductivity (EC) was determined using a DDSJ-308A conductivity meter (Leici, Shanghai, China). Soil salt content was calculated according to the method of Bao [76]:

Soil salt content (mg kg^−1^) = EC × 0.064 × 5 × 10 × 1000

(3) Cation exchange capacity and exchangeable sodium

Cation exchange capacity (CEC) was determined by the hexamminecobalt(III) chloride ([Co(NH_3_)_6_]Cl_3_) extraction–spectrophotometric method. Exchangeable sodium was measured by the NH_4_OAc–NH_4_OH flame photometry method [75]. The exchangeable sodium percentage (ESP) was calculated as:

ESP (%) = (Exchangeable Na^+^/CEC) × 100%

(4) Soil nutrient contents

Soil organic matter content was determined by the potassium dichromate external heating method. Available nitrogen was measured by the alkaline hydrolysis diffusion method. Available phosphorus was extracted with 0.5 M NaHCO_3_ and determined spectrophotometrically. Available potassium was extracted with CH_3_COONH_4_ and measured by flame photometry. All of the above nutrient determinations followed the standard procedures described by Bao [76].

#### 4.3.2. Alfalfa Overwintering Rate

Overwintering rate: At the withering stage (15 October 2023), three 1 m segments were randomly selected in each plot, and the plants within each segment were marked. After regrowth in the following spring, the number of surviving marked plants was recorded, and the overwintering rate was calculated as:

Overwintering rate (%) = (Number of surviving plants/Number of marked plants before winter) × 100%.

#### 4.3.3. Alfalfa Productivity

Growth measurements (15 June 2024, initial flowering stage):

SPAD value: The relative chlorophyll content (SPAD) of the third functional leaf from the apex of the main stem was measured using a SPAD-502 chlorophyll meter (Konica Minolta, Tokyo, Japan.

Photosynthetic parameters: Net photosynthetic rate (Pn, μmol m^−2^ s^−1^), stomatal conductance (Gs, mol m^−2^ s^−1^), and intercellular CO_2_ concentration (Ci, μmol mol^−1^) were measured between 10:00 and 12:00 using a Li-6400 portable photosynthesis system (LI-COR, USA).

Plant height: At the initial flowering stage, ten uniformly growing alfalfa plants were randomly selected from each plot, and the vertical height was measured and averaged.

Branch number: At the initial flowering stage, the number of alfalfa branches was counted within a randomly selected 1 m^2^ quadrat in each plot.

Stem-to-leaf ratio: During yield measurement, ten uniformly growing plants were randomly sampled from each plot. The stems, leaves, and inflorescences were separated (inflorescences were grouped with leaves). Samples were oven-dried at 105 °C for 15 min, then at 70–80 °C to constant weight, and weighed separately. The stem-to-leaf ratio was calculated as:

Stem-to-leaf ratio = Stem dry weight/Leaf dry weight.

Hay yield: Only the first cutting yield was measured. The entire plot was harvested at a stubble height of approximately 5 cm. Fresh weight was recorded, and a 500 g subsample was collected, oven-dried at 105 °C for 30 min, then at 70–80 °C to constant weight, and weighed to calculate the fresh-to-dry ratio and dry matter yield.

### 4.4. Statistical Analysis

Data preprocessing was performed using Microsoft Excel 2021 (Microsoft, Redmond, WA, USA). Statistical analysis was performed using SPSS Statistics 26.0 (IBM, Armonk, NY, USA). One-way analysis of variance (ANOVA) combined with Duncan’s multiple range test and the LSD test was used to assess differences among treatments, with the significance level at *p* < 0.05. All figures were plotted using Origin 2021 (OriginLab, Northampton, MA, USA).

To systematically quantify the causal pathways linking mulching management practices, soil conditions, crop physiological traits, and forage production outcomes, partial least squares structural equation modeling (PLS-SEM) was employed using the plspm package in R (version 4.3.1) [77]. Based on a soil–plant–production theoretical framework, mulching density (CK, LC, MC, and HC) was set as the exogenous variable, while five latent constructs—soil physical improvement, soil chemical amelioration, soil nutrient enrichment, plant physiological enhancement, and plant morphological development—served as endogenous mediating variables influencing forage yield and quality. Reflective measurement models were specified for all latent constructs. Path relationships were tested using path coefficients (β), with significance assessed via bias-corrected bootstrap confidence intervals (5000 resamples). Measurement reliability and validity were evaluated using standardized outer loadings, Cronbach’s alpha, composite reliability (CR), and average variance extracted (AVE); discriminant validity was assessed using the heterotrait–monotrait ratio of correlations (HTMT). In addition to the GoF index, model fit was evaluated using the standardized root mean square residual (SRMR), with values below 0.08 considered acceptable. Potential common method bias was assessed using the full collinearity test, with variance inflation factors (VIF) below 3.3 considered acceptable. Given the modest sample size (*n* = 12 plots), all inferential interpretations relied on bootstrap confidence intervals. Model explanatory power was assessed via the coefficient of determination (R^2^). Overall model goodness of fit was evaluated using the GoF index, defined as the geometric mean of average communality and average R^2^; according to Tenenhaus et al. [77], GoF values of 0.10, 0.25, and 0.36 represent weak, moderate, and strong model fit, respectively. Additionally, a comprehensive radar chart analysis was conducted to evaluate the integrated soil amelioration effect by standardizing all soil indicators to a 0–1 scale.

### 4.5. Generative Artificial Intelligence Assistance

During the preparation of this manuscript, Deepseek-V3 was used to assist in sorting out the logical framework of the research background in the Introduction section and polishing the academic language expression of the Discussion section. All AI-generated content was carefully reviewed, revised and validated by all authors to ensure its accuracy and consistency with the experimental data. The authors take full responsibility for the originality, accuracy and integrity of the entire manuscript content.

## 5. Conclusions

This study demonstrates that grass clipping mulching effectively ameliorates soda saline–alkali soil and enhances alfalfa productivity through a multi-level cascade mechanism. High-density mulching (18,000 kg ha^−1^) significantly reduced soil pH, electrical conductivity, and ESP, while increasing water content, porosity, and organic matter. These soil improvements significantly promoted alfalfa growth, photosynthesis, and overwintering survival, and simultaneously enhanced both hay yield and forage quality. PLS-SEM modeling confirmed that mulching density primarily drives soil nutrient enrichment, which subsequently enhances plant physiology and ultimately promotes forage yield and quality, with robust model fit (GoF = 0.76). These findings establish a mechanistic framework for organic mulching in saline–alkali soil remediation and highlight the dual agronomic and economic benefits of utilizing rain-damaged, moldy grass clippings as a soil amendment. Future research should focus on long-term microbial dynamics and optimized mulching rates for cost-efficient field application.

## Figures and Tables

**Table 1:** Effects of grass clipping mulching on forage quality of alfalfa in the second year on soda saline–alkali soil.

Treatment	CP (%)	CF (%)	ADF (%)	NDF (%)	RFV
CK	18.87 ± 0.45 b	3.12 ± 0.04 c	31.72 ± 0.34 a	37.69 ± 0.26 a	158.43 ± 1.64 c
LC	19.47 ± 0.13 b	3.21 ± 0.05 bc	30.75 ± 0.26 ab	37.57 ± 0.90 a	160.89 ± 4.00 bc
MC	19.84 ± 0.28 a	3.59 ± 0.07 a	29.21 ± 0.77 b	37.43 ± 0.74 a	164.41 ± 1.77 b
HC	20.42 ± 0.28 a	3.53 ± 0.05 ab	28.20 ± 0.89 c	37.27 ± 0.89 a	167.08 ± 2.57 a

Note: CK, no mulching; LC, low-density grass clipping mulching; MC, medium-density grass clipping mulching; HC, high-density grass clipping mulching. Values are means ± standard deviation (SD, *n* = 3). Different lowercase letters within a column indicate significant differences among treatments at *p* < 0.05 by Duncan’s multiple range test. Relative feed value (RFV) was calculated based on acid detergent fiber (ADF) and neutral detergent fiber (NDF) according to standard forage evaluation protocols.

**Table 2:** Soil physical and chemical properties of the experimental site.

Soil Type	pH	EC (mS cm^−1^)	CO_3_^2−^ (mg kg^−1^)	HCO_3_^−^ (g kg^−1^)	Cl^−^ (g kg^−1^)	Na^+^ (g kg^−1^)	SOM (g kg^−1^)	Available N (mg kg^−1^)	Available P (mg kg^−1^)	Available K (mg kg^−1^)
Saline–alkali soil	8.90	0.22	2.61	0.81	0.32	1.63	9.4	98.34	11.07	56.42

Note: EC, electrical conductivity; SOM, soil organic matter.

**Table 3:** Grass clipping mulch application rates and treatment codes.

Treatment	Grass Clippings Rate (kg ha^−1^/t ha^−1^)	Code
No mulch	0 (0)	CK
Low-density mulch	6000 (6.0)	LC
Medium-density mulch	12,000 (12.0)	MC
High-density mulch	18,000 (18.0)	HC

Note: Rates are presented on an as-applied (air-dry) mass basis with 14% wet-basis moisture content. Corresponding dry-matter application rates are 0, 5.16, 10.32, and 15.48 t ha^−1^ for CK, LC, MC, and HC, respectively.

## Data Availability

All data generated or analyzed in this study are included in the article.
